# Supramolecular systems and their connection with metal–organic structures

**DOI:** 10.3389/fchem.2024.1468916

**Published:** 2024-11-05

**Authors:** Rodrigo Cué-Sampedro, José Antonio Sánchez-Fernández

**Affiliations:** ^1^ School of Engineering and Sciences, Monterrey Institute of Technology, Monterrey, Nuevo León, Mexico; ^2^ Department of Polymerization Processes, Center for Research in Applied Chemistry, Saltillo, Coahuila, Mexico

**Keywords:** supramolecular assemblies, metal–organic framework, switchable MOFs, self-assembled structures, metalloporphyrins, confined space

## Abstract

Supramolecular structures with specific applications are a pillar in several areas of science. Thus, from a contemporary point of view, there are several reasons to embrace a systematic order of the supramolecular concept itself. First, the structuring of a supramolecular material seems safer now than it did decades ago. Second, the interactions of metal–organic frameworks (MOFs) and supramolecular chemistry and, conversely, supramolecularity to assemble MOFs and create efficient complex systems in multiple cutting-edge applications are an image to be safeguarded. Third, perhaps we should simply limit ourselves to considering how researchers in these fields have attempted to correlate the notion of supramolecular systems by linking self-assembly considerations. In any case, these topics present advantages to optimize innovative geometries that are useful to highlight significant practical applications. This review covers a general introduction to MOFs and supramolecularity, the key unit of the study presented here, followed by a survey of recent advances in confined space chemistry, the relationships of MOFs with supramolecular structures, and the synthesis electrochemistry of MOFs and switchable MOFs to obtain a greater understanding of structure–property relationships. To conclude, some future perspectives on this promising and plausible field of science will be mentioned.

## 1 Introduction

The varied sizes, shapes, and charge distributions of supramolecular systems provide an interesting set of challenges for co-design with other structures to enhance their applicability. This situation requires, as does the high range of molecular architectures, the visualization of diversified chemical nuances of scientific knowledge.

By determining the structure, properties, and interactions of molecules, primary chemical forces are of immense importance for chemical and biological processes. Consequently, material sciences, organocatalysis, structural chemistry, structural biology, and supramolecular and pharmaceutical chemistry must be adapted in a fine context, for instance, a systematic link with metal–organic frameworks. Chemical forces such as the hydrogen bond, the London dispersion, the dipole–dipole, and the π‒π interactions have received close attention and generated a considerable body of literature. The present article is centered around macromolecular and supramolecular structures and their interaction with structures built under non-traditional schemes.

**FIGURE 1:**
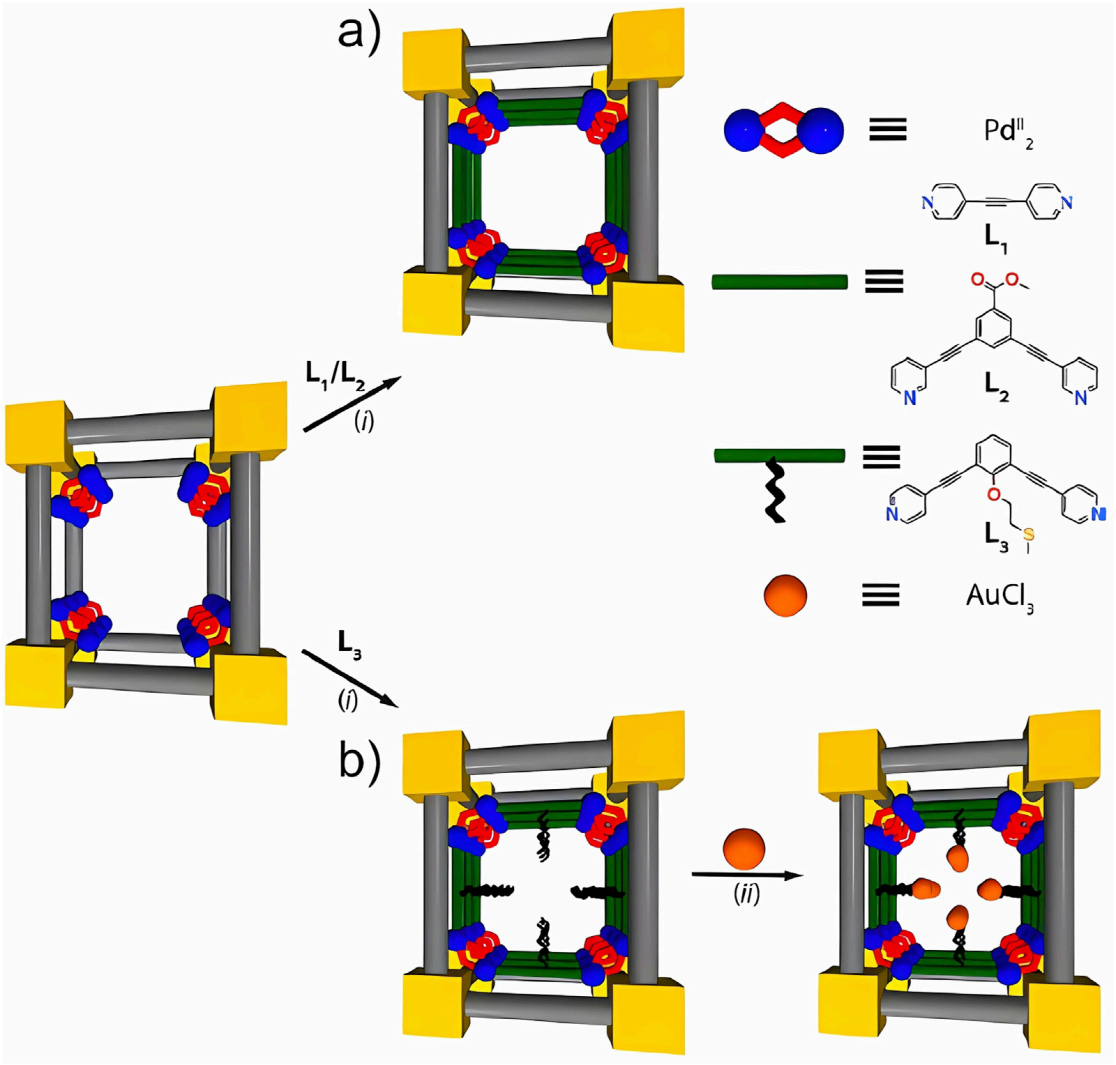
Sequential synthesis of homo- **(A)** and heterobimetallic **(B)** mechanically bonded, catalytically active SCCs within a confined space into the MOF channels (SCCs@MOFs). (i) Incorporation of the desired organic ligand with suitable encoded structural and coordination information and (ii) post-assembly metalation of preformed SCCs@MOFs. Adapted from Adam et al. (2019).

The incursion into self-assembly issues to create structures with defined supramolecular architectures has given rise to strategies to obtain organometallic structures such as a homogeneous supramolecular metal–organic framework from a hexa-armed precursor based on [Ru (bpy)_3_]^2+^ and cucurbit (8) uril (Tian et al., 2016). The possible variety of these compounds has been visualized to develop a comprehensive work using *in situ* heterogeneous self-assembly and structural characterization by single-crystal X-ray diffraction (SCXRD) of mechanically bonded supramolecular coordination compounds (SCCs) within a confined space of MOF channels (SCCs@MOFs). Specifically, the structures consist of a Pd_8_ square metal‒organic polygon, a discrete Pd_16_ supramolecular cage, and a heterobimetallic Au^III^–Pd^II^ cage (Adam et al., 2019). While the cooperative effect clearly plays an important role between the available void space and the distance between Pd_2,_ including the accessibility of Pd^II^ ions in the MOF [Pd^II^(NH_3_)_4_][Pd^II^_2_(*μ*‒O) (NH_3_)_6_) (NH_4_)_2_]_0.5_{Ni^II^_4_ [Cu^II^_2_(Me_3_mpba)_2_]_3_}·52H_2_O with linear L_1_ and bent L_2,3_ ligands, it can promote the formation of self-assembled SCCs within the confined space of MOF channels, as depicted in Figure 1 (Adam et al., 2019). Supramolecular chemistry has broadened discussions about the applications of host–guest systems, such as molecular recognition, drug delivery, stereoselective organic synthesis, catalysis, and bioactive materials. The robustness to form supramolecular interactions with diverse guest molecules at specific sites, such as hydrogen bonding, electrostatic interactions, π–π interactions, and hydrophobic effects, has been commented on by Liu et al. (2023).

The underlying mechanisms were described in the review work by Noor. He realizes the relationship of sterically bulky ligands with the chemical reactivity due to the fine-tuning of electronic effect in ligands designed as an important part that can restrict the intrinsic reactivity (Noor, 2023). According to Horiuchi and Umakoshi (2023), luminescence is an important aspect of studying the metal–metal interactions in heteropolynuclear complexes because it is sensitive to small changes in the energy differences between ground and excited states. They focused on the supramolecular chemistry and noncovalent interactions of metal complexes with coordinated N‒H pyrazoles, taking advantage of the fact that pyrazoles are weak acids and act as both σ-donor and π-acceptor ligands. Due to the above, the authors delve into recent advances on synthetic, structural, thermodynamic, electronic, and photophysical properties of Pt- and Pd-based heteropolynuclear complexes.

Wang et al. (2021) verified that the link between MOFs and MOGs offers a wide selection of combinations connecting metal nodes and organic linkers to produce electrocatalysts with high surface areas, variable porosity, and excellent activity after pyrolysis. In oxygen electrocatalysis studies using MOFs and MOGs, a correlation has been established between oxygen catalyst performance and the intrinsic structure, active-site density, surface property, charge conductivity, and mass transport.

Several studies from the literature survey the self-assembly of MOF particles into ordered superstructures of different dimensions. The dynamic behavior for creating ordered MOF superstructures, including solvent evaporation, depletion-assisted assembly, electric field-assisted assembly, DNA-assisted assembly, anisotropic pattern-assisted assembly, ice-templated self-assembly, and air–liquid interface assembly, has been the main aim and concern of several researchers (Fonseca et al., 2023). Supramolecular gels comprise an intriguing contemporary research field with current and future challenges. Dynamic development of techniques as NMR, SEM, XRD, and others has opened new facets in the study of supramolecular gels and their properties (Kuosmanen et al., 2020).

Metal oxide node hydroxyl groups are formed during the synthesis of MOFs by hydrolysis of metal precursors, so the hydroxyl groups are incorporated into the metal oxide clusters that are nucleated in the aqueous reaction medium. These groups become structural ligands, such as the *μ*_3_‒OH groups bonded to Zr_6_O_8_ nodes and the *μ*_2_–OH groups bonded to Al–OH–Al nodes. On the other hand, MOF node hydroxyl groups can be generated by reactions of reactive node sites or ligands with water in post-synthesis treatments (Yang and Gates, 2024). Similarly, Zr-based clusters with open environments around the secondary building units (SBUs) make the metal node-supported cobalt catalysts more active in the hydrogenation. Therefore, as pointed out by Lin and coworkers, advanced Zr_8_ (*μ*_2_–O)_8_ (*μ*_2_–OH)_4_ MOF nodes with sterically open Zr_2_–*μ*_2_–OH ligand sites support Co-based catalysts for the hydrogenation of a broad range of unsaturated compounds such as olefins, imines, carbonyls, and heterocycles (Ji et al., 2016). For instance, metallic node-bound acetate ligands were stable at temperatures up to 200°C, while they were eliminated substantially by evacuation at 250°C; these findings are consistent with observations for acetate ligands on the Zr_6_O_8_ nodes of UiO-66. The reactivities of the node ligands were probed with a flow of methanol vapor at 80°C and 1 bar for 24 h and then evacuated at 80°C for 12 h to remove any physiosorbed species (Xiao et al., 2021; Kandiah et al., 2010). In another case, the same criteria of manipulation of cations, linkers, and solvents were expanded to Ln-DXTA MOF (where X = Cl and Br) structures with specific network topology and supramolecular interactions. In this regard, researching dihalogenated terephthalates explores the coordination chemistry of Ln^III^ with 2,5-dihaloterephthalate (Ln-DXTA) ligands (Smith et al., 2024).

Alternatively, Arcudi and Đorđević point out that any noncovalent interaction approach satisfactory for preparing carbon-dot-based assemblies and composites can leverage the features of supramolecular chemistry, including responsiveness to stimuli, due to the dynamic nature of noncovalent interactions. In short, coordination complexes are fundamental for any metal–ligand interactions for customized combinations toward structuring carbon-based dots (Arcudi and Đorđević, 2023).

As described, the relationships of the organometallic structures with the supramolecular essence with a considerable mixture of metals allow a variety of functions limited by stiffness with variable stress relaxation rates. This fundamental scheme can be expanded onto the supramolecular polymer networks (Lundberg et al., 2024).

Note that the related spaces between organometallic networks and supramolecular details are increasingly smaller. Likewise, it is necessary to emphasize that due to the directionality of metallic nodes and ligands, the geometry of the synthons involved in the noncovalent interaction is translated into the geometry of the final supramolecular structure.

## 2 Supramolecular chemistry in confined space

Among the molecular systems that present internal cavities typical of confined inner cavities, we can mention crown ethers, cryptands, molecular boxes, pillararenes, cucurbiturils, and other macrocycles and precisely display their specific chemical reactivity. Due to their structural attributes, MOFs are an excellent host for confinement and are an outstanding heterogeneous catalyst model (Begum et al., 2020).

In contrast, there are steric limitations in the cavities of MOFs and their walls formed by reactive structures where components are housed despite the unfavorable steric factors adopting very specific conformations. Several reports have described that the steric constraints, solvent exclusion, intermediates stabilization, and conformational control of substrates provide a strong impetus for chemical reactions in a confined space as an alternative for unconventional processes (Iuliano et al., 2023). In this regard, significant details established by supramolecular chemistry incorporate molecular recognition, host–guest chemistry, molecular self-assembly, folding, and mechanically interlocked molecular architectures that presuppose dynamic covalent chemistry. Depending on geometries, support not only stabilizes the catalytic site but must also offer synthetic flexibility to tune the chemical environment beyond the coordination spheres based on the incorporation of molecularly defined catalytic functions into MOFs (Suremann et al., 2023). In this respect, the diffusion within a MOF pore may be radically different in homogeneous or nonconfined heterogeneous systems; this obviously affects the kinetics of a specific reaction. The molecules near MOF particles move according to the bulk molecular diffusivity and the motions of the fluid to regulate the flow reactions (Iliescu et al., 2023). For their part, Lin and collaborators mention the coverage of a 2D COF based on spirobifluorene blocks for dual photoredox and Ni catalysis (Fan_2023). At the same time, studies by Guo Q. Y. et al. (2023) mentioned the synthesis of a reusable catalyst by stabilizing dinickel active sites using the bipyridine groups in MOF-253 with the formula of Al(OH) (2,2′-bipyridine-5,5′-dicarboxylate) for *Z*-selective semihydrogenation of alkynes and selective hydrogenation of C=C bonds in α,β-unsaturated aldehydes and ketones.

Referring specifically to low-symmetry ligands that do not contain coordinating units of different denticities, Lewis and Crowley indicate an alternative mode for controlling the self-assembly process that must be incorporated into the structure. In addition, the directionality of lone pair orbitals and the predictable coordination geometry of transition-metal ions add a geometric hindrance to ligand frameworks. In turn, they avoid the formation of isomeric mixtures during metallo-supramolecular self-assembly (Lewis and Crowley, 2020).

The development of supramolecular and reticular chemistry converges in the encapsulation in nanoconfined spaces. This connection, which maintains its roots in classical host–guest chemistry, is the origin of encapsulation in the porous network of the MOFs. Encapsulation occurs when functional species are held upon the walls of an MOF by either covalent bonding or van der Waals interactions in such a way that they cannot leach due to their stabilization and geometric factors. The outcome based on each nanospace of MOFs engendered by the coordination nanospace remains better interpretable about the discrimination of guest molecules and also enables the synthesis and conversion of value-added substances by virtue of nanomodulated reaction design in the confined space (Hosono and Kitagawa, 2018). For instance, a representative and particularly interesting nanoreactor offers a limited, confined space that imprints a key role in the chemical process by controlling the number of molecules inside and their noncovalent forces, which are predominantly guided by the supramolecular chemistry and their dynamic molecular interactions (Bruno et al., 2023).

In comparison to typical covalently connected organic supramolecular hosts, metal–organic capsules are spontaneously generated by simply mixing modular building units, consistent with the general trend in pre-organized ligands’ ability to produce symmetrically predesigned structures. In that respect, Jing et al. established strategic guidelines to build photoactive supramolecular systems by encapsulating dye molecules within the inner space of redox-active hosts. In this view, photochemistry demonstrated advantages due to the particular structural confinement, avoiding excited-state quenching caused by other chemical species concerning the radical intermediate complex by adjusting the absorption or emission of the guest through the transfer of electrons that are translated into energy (Jing et al., 2019).

In good agreement with Sinha and Mukherjee, the cavity size for absorption of the substrate and easy release of the product made using supramolecular architectures a better choice for enzymatic catalytic transformation. The enzymatic interactions in supramolecular molecules consolidate the conformational organization and compartmentalization by a self-assembled coordination complex (Sinha and Mukherjee, 2018).

**FIGURE 2:**
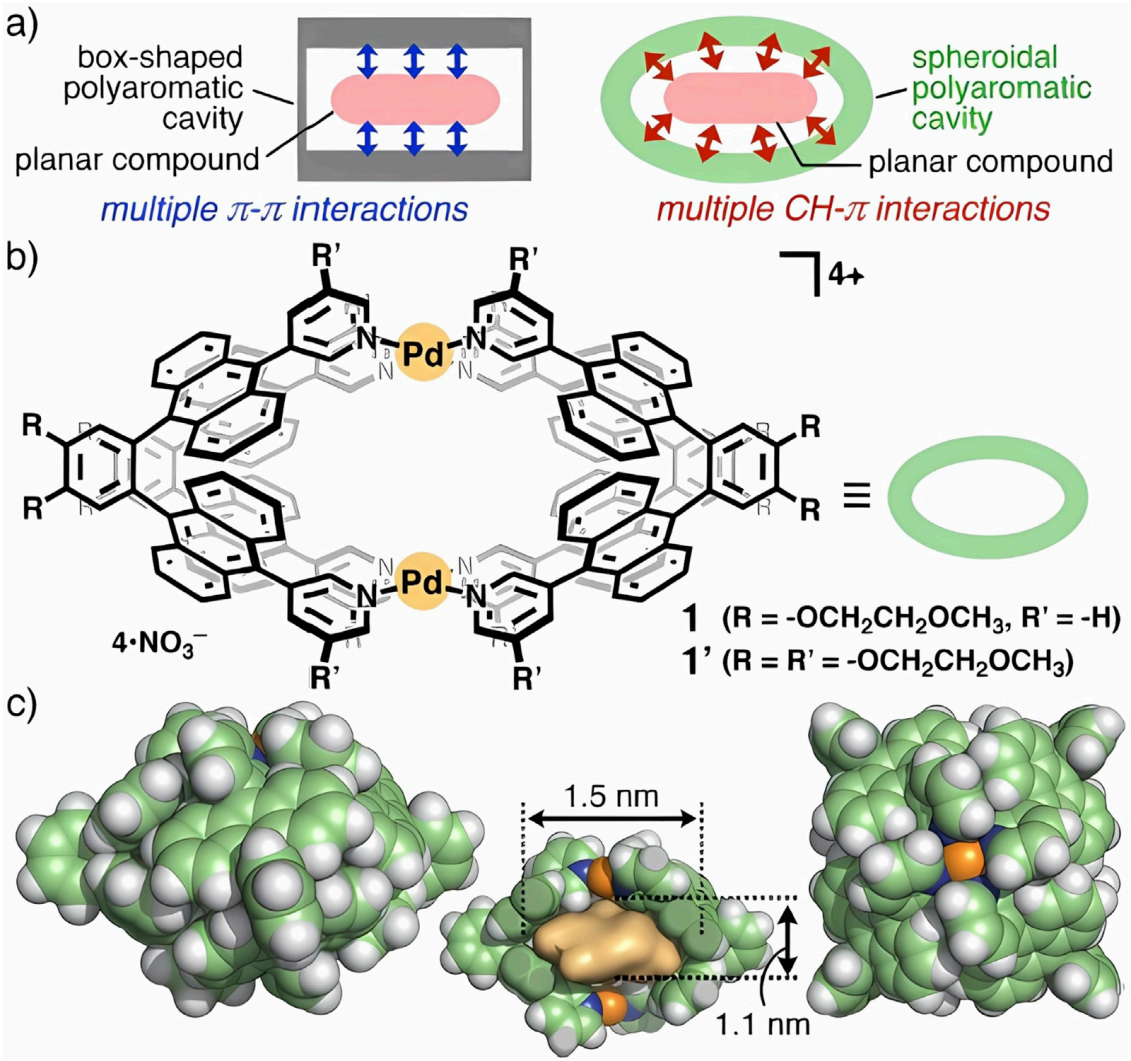
**(A)** Multiple host-guest π–π and CH–π interactions in box-shaped and spheroidal polyaromatic cavities. **(B)** Spheroidal polyaromatic capsules 1 and 1′, and **(C)** the crystal structure (side, sliced side, and top views; R = R′ = H. The inner cavity is highlighted in yellow. Adapted from Kishida et al. (2022).

Lately, the group of Yoshizawa has conducted a conceivable host–guest system using CH–π interactions employing a polyaromatic capsule with an atypical spheroidal cavity. The observations demonstrated that a perfectly structured cavity efficiently binds each molecule of planar compounds through multiple CH–π interactions rather than π–π interactions. Complex mixtures of polyaromatic molecules provide 12 and 10 aromatic CH groups. The authors detail that a selective binding of planar polyaromatics and a planar metal complex utilizing multiple CH–π interactions may be the key to providing novel insight into molecular chemical interaction bonding explored using multiple polyaromatic compounds and the porphin Cu^II^ complex or bis(acetylacetonato) framework and their diverse structural affinity (Kishida et al., 2022). Rearrangement of molecular addends can easily be determined if they are accompanied by a change in the pattern of a polyaromatic cavity in addition to metallic coordination, leading to a spheroidal shape characterized by multiple CH–π interactions, such as is shown explicitly in Figure 2A (Kishida et al., 2022). For this reason, coronene and pyrene molecules were selected to design spheroidal capsules, as shown in Figures 2B, C (Kishida et al., 2022). Although the incorporation of multiple interacting groups to achieve high guest affinity has been a key principle of supramolecular chemistry for decades, it is only recently that phenyl unions, halogen bonds, and tetrahedrally shaped adamantane [(1,3,5,7-tetrakis (4-bromophenyl) adamantane] have been the subject of study in the modulation of channels that can confine aromatic petrochemicals and chloroform. The supramolecular framework can, via van der Waals contacts, confine solvent molecules predominately using π‒π interactions, which could make the efficient separation of toluene from benzene possible (Lutz et al., 2024).

Another contribution exhibiting a confined chiral microenvironment has proven very efficient in engineering such host–guest interactions and a reactive conformation exerting control over the chemo-, regio-, and stereoselectivity of asymmetric conversion, resulting in considerable activity compared with the bulk solution. In addition, based on noncovalent interactions, the cavities of cages could stabilize reactive and unstable species, promote the transformation of high-energy intermediates, and minimize side reactions linked to the transition states that can occur (Tan et al., 2019). Adapting certain tailored properties of a crystalline solid by controlling its structure and composition at the atomic level presents distinctive opportunities for the design of new functional materials. Strategies employed have given rise to low-dimensional coordination polymers formed by linking Re_6_Se_8_ superatomic cores and applying bipyridine bridging ligands and a carboxylic-acid-based synthetic strategy to create 2D and 3D MOFs with Co_6_Se_8_ superatoms (Doud et al., 2020). It is worth mentioning at this point that the preparation of 3D mesostructures obtained by the crystallization-driven self-assembly (CDSA) of coil-crystalline block copolymers (BCPs) studied by Guerin et al. ensured a strong attractive intramolecular interaction. The composition of the solvent is decisive in determining the structural sequences, from closed 3D multi-tori spherical shells to 2D toroid mesh monolayers. Explicitly, the self-assembly of polyferrocenyldimethylsilane-block-polystyrene (PFS_26_-b-PS_306_, with *M* ^PS^ ≈ 30,000) can be favored using an acetone/decane mixture with a 25% volume of acetone. The PFS26-b-PS306 microphase separation can be predicted based on variations in the solvent mixture (Guerin et al., 2020).

## 3 Supramolecular assemblies

Lehn (2013) conceived the term supramolecular chemistry as a field of science based upon molecular chemistry; he also marked the foundations of self-organization processes and adaptative chemistry under the premise of constitutional dynamic chemistry (CDC) with the explicitness of being associated at the molecular level under a covalent dynamic and in a supramolecular architecture constituted by a noncovalent dynamic. In any case, using dynamic chemistry leads us to control supramolecular chirality in a systematic way. As indicated by Wang et al. (2024), the N-protected fluorinated phenylalanine can present several switchable states by manipulating chirality and forming achiral spherical nanoarchitectures. After protonation, chiral expression is feasible at the macroscopic level.

All supramolecular structures are designed with a self-assembly technique and can interact with biological systems; therefore, they can be used as drug delivery systems (Tu et al., 2016). In self-assembly, coordination systems with metal atoms can generate supramolecular materials that are too complex (Jiang et al., 2016). This also implies the principles of symmetry that provide some very insightful tools for reaching an understanding of many aspects of the chemical and physical world. This characteristic can be established through covalent interactions, hydrogen bonds, and van der Waals forces without excluding other inter- and intramolecular interactions (Wu et al., 2018). In an alternative approach, Nitschke and coworkers (McTernan et al., 2022) point out that the design of metal–organic architectures is a function of directional strategies described as the directional-bonding approach, the symmetry interaction, the molecular paneling outlook, and the weak-link perspective. The success of these strategies has been employed to predict metallomacrocycle-based, high-symmetry three-dimensional architectures.

The incorporation of multiple complex systems in MOFs, such as the introduction of multiple metals, implies structural difficulties, so in this line, atom probe tomography (APT) can provide useful information in characterizing mixed-metal rod MOFs (Ji et al., 2020). Many of the applications of the MOFs have their beginnings in APT and can be developed basically by a sequence of metals. This illustrates the novelty of regulated targets for generating metal sequences into a mixed-metal rod MOF. This, in turn, involves thermodynamic considerations that are noticeable in a random sequence. Primarily, it maximizes configurational entropy that could be translated into the minimum free energy under the assumption that the enthalpy change involved in the permutation of components is negligible. In contrast, this assumption cannot be true if metals of varying sizes are mixed into an alloy (Ji et al., 2020).

The noncovalent bases of these systems are the cause of extraordinary structural change as a consequence of variations in temperature, pH, and ionic strength (Insua et al., 2022), which could not happen with systems built by covalent interactions. Supramolecular assembly systems are built through several reversible weak interactions to form structures with well-defined architectures. Supramolecular chemistry plays with dynamic noncovalent interactions, including hydrogen bonding, host–guest interactions, π–π stacking, metal coordination, hydrophobic forces, and electrostatic effects (Huang and Ma, 2020). The relative importance of the supramolecular assembly system is significant in developing optical materials with supramolecular tools. Considerable progress has been made in the field of assembled luminescent materials. This is one reason for reinforcing investigations on polymeric supra-amphiphile topologies (Chang et al., 2019), as well as the photoactive supramolecular functionality for light harvesting (Chen et al., 2023).

The design of a topological supramolecular network is an attractive bioelectronics task, such as designing biocompatible conducting polymers (CPs) with high conductivity and high stretchability. The product must be supported after microfabrication and designed to ensure the non-formation of cracks under 100% strain, which is essential for the maintenance of low-impedance and seamless biointegration (Jiang et al., 2022).

**FIGURE 3:**
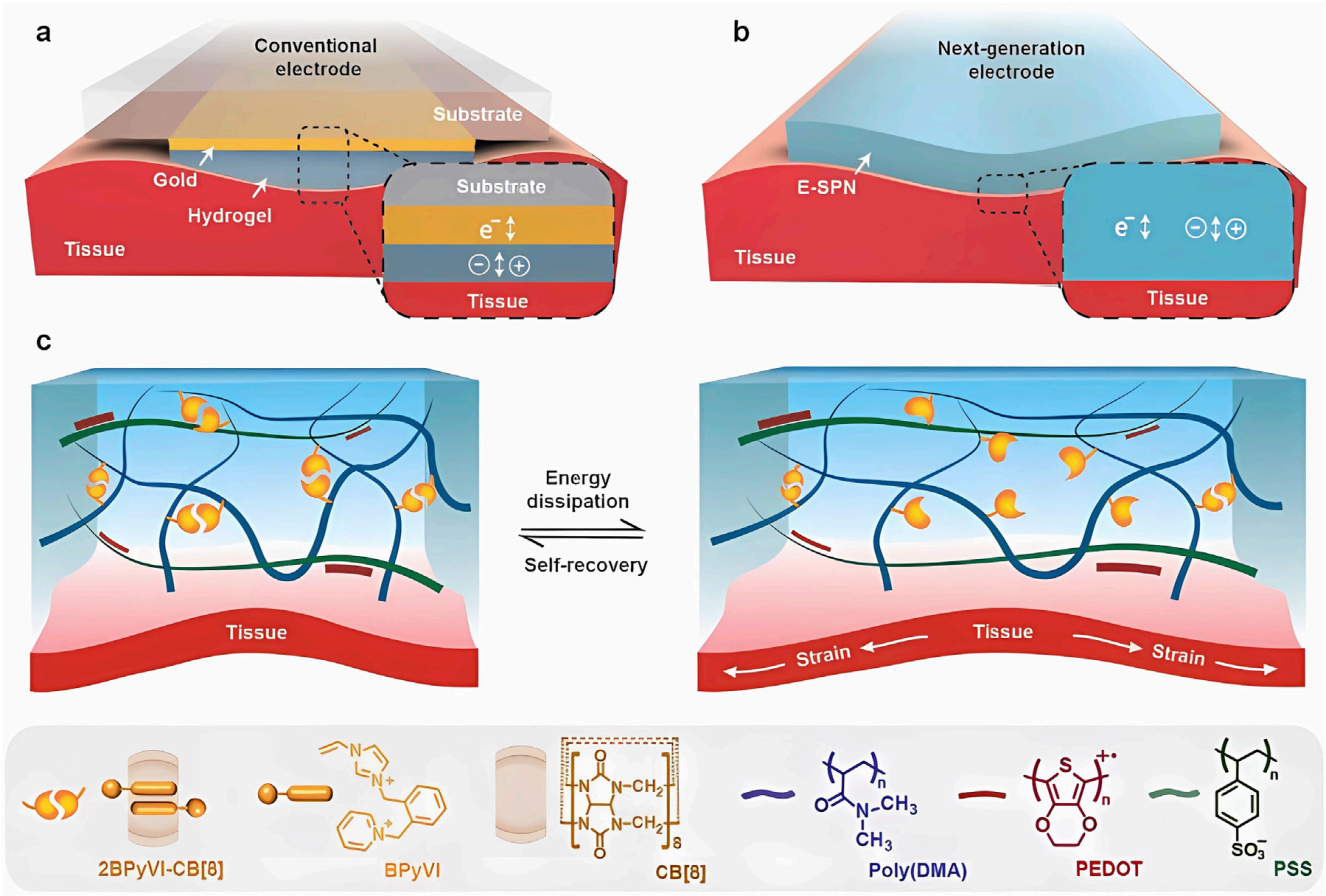
Design of E-SPN bioelectrode. **(A)** Schematic of conventional wearable electrodes using hydrogels with conventional ionic conductivity, requiring metal and a substrate. **(B)** Schematic of a next-generation, stretchable bioelectrode with dual ionic and electronic conductivity. **(C)** Schematic of the E-SPN material design with molecular structures. CB [8] supramolecular ternary complexes are dynamic crosslinks that rupture under deformation to dissipate energy and can be reformed, resulting in a highly deformable supramolecular network with self-recovery. Adapted from O’Neill et al. (2023).

Scherman’s research team established an electrically conducting supramolecular polymer network (E-SPN) by incorporating PEDOT:PSS within a dynamic CB [8]-cross-linked poly (dimethylacrylamide) network. The product displayed an electrical conductivity (52 S m^−1^) and achieved supramolecular tissue mimicry with low Young’s modulus, high compressibility, stretchability, toughness, high water content, and rapid self-recovery. Consequently, the E-SPN can maintain an unmatched mechanical-electronic relationship with PEDOT:PSS hydrogels (O’Neill et al., 2023) (see Figure 3C). The well-known role played by aqueous supramolecular solutions used as lubricants relies on polymer and lamellar solutions at the molecular scale, which are in the boundary lubrication regime regarding their friction mechanisms. This could be true in molecules such as polyalkylene glycol (PAG), which is composed of ethylene oxide (EO) and/or propylene oxide (PO) units and is used to control the sol/gel transition of aqueous solutions (Asada et al., 2022). Conventional bioelectronic devices contain hydrogels either in the interfacial layer or forming a coating between the tissue and metallic conductor component, as seen in Figure 3A. Hydrogels may also form stretchable standalone bioelectrodes (see Figure 3B).

Seiffert and collaborators carried out high-value work using transitory bonds in metal–ligand complexes. They functionalized tetra-arm poly (ethylene glycol) (tetraPEG) precursors with 2,9-dimesityl-1,10-phenanthroline to form networks in combination with either phenanthroline or terpyridine ligands at 1:1 ratios. Supported by reliable results, they suggested that the coordination geometry preference of the utilized metal ion in comparison with the geometry requirements of the desired heteroleptic complex can be linked to defects in the composition (Ahmadi et al., 2023).

In the structural design and construction of supramolecular materials, it is well documented that the π stacking interaction can induce a narrow bandgap in the MOFs. Important observations can be drawn in this regard for the tetrathiafulvalene (TTF) and MIL-140A families (Butova et al., 2020), where the width of the bandgap decreases as the interaction distance shortens and the interaction energy is enhanced. The functional groups that induce polarity in aromatic rings promote supramolecular electrostatic coupling. The formation of donor–acceptor systems through the addition of functional groups is a reliable strategy to promote highly efficient π–stacking interactions, where much narrower bandgaps can be evident (Alfonso Herrera and Beltrán, 2024).

There are only a few examples of structural design showing the involvement of halogen bonding by using pyridine in nonaqueous media to stabilize the phosphomolybdate and the six terminal ligand sites within the structural vacancy between oxide and pyridyl ligands. On the one hand, the structure is controlled by intramolecular spatial separation of coordinated halogenated ligands, that is, intermolecular halogen–halogen interactions in the crystalline state, and on the other, it is a useful tool to study the design of geometrical supramolecular materials, considering halogen–halogen interconnections (Choudhari et al., 2024).

**FIGURE 4:**
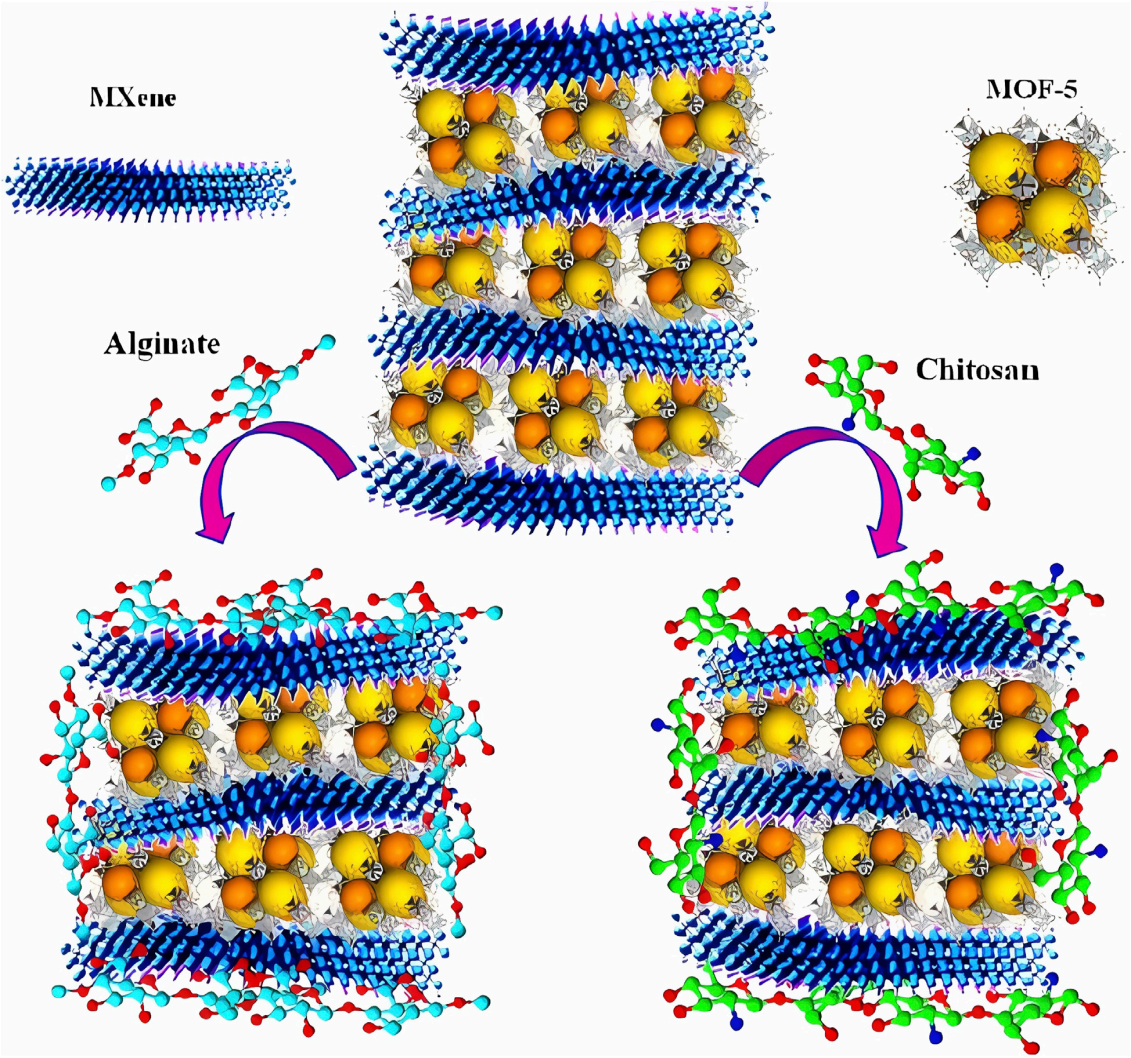
Illustrative representation of MXene/MOF-5 and its alginate and chitosan nanostructures. Adapted from Rabiee et al. (2021).

Functionalization of perylene bisimide (PBI) with styrene moieties allows leveraging a combined supramolecularity and photopolymerization to improve the photosynthetic architecture of artificial quantasomes based on PBI in terms of lateral and orthogonal lamellar fixation. It should also be mentioned that optimized geometries obtained depend, usually in a systematic way, on the basis set and level of the formation of the quantasome assembly, with lateral aggregation favored by the proximity of a polymerizable styrene. It is plausible that the addition yields up to 290% compared to the styrene-free reference. A perfectly designed structural architecture of the PBI-constituted chromophore can be established due to the synthesis of a biscationic PBI scaffold with two terminal styrene groups Figure 4 can be established. This gives rise to the formation of a quantasome assembly in water (QS-S), favoring the proximity of the polymerizable styrene groups with high local density (Ranscht et al., 2024).

One criticism of the bonding patterns in supramolecular assemblies is that they inherently lead to high-symmetry products. In response, there has been a concerted effort to produce low-symmetry metal–organic cages using various tactics, including steric, geometric complementarity, and interactions between the ligands. While model compounds do not display optimized properties, their assembly shows promising perspectives toward the design of supramolecular frameworks, showing a good relationship with MOFs.

## 4 Significant advances in MOF architecture and its analogs assembled by novel strategies

Considerations of the geometric requirements for a target framework and implementation of the design are called reticular synthesis. This operation requires an understanding of the coordination patterns of the metal and organic units that are the source of the formation of ideal topologies. The reliability of the conclusions drawn from those assertions confirms what some authors have emphasized about MOFs consisting of tetrahedral Zn^2+^: they are not chemically stable. In contrast, systems that contain Zn^2+^ in a different coordination setting, such as MOF-69 that contains both tetrahedral and octahedral Zn centers that share oxygen, have shown more stability than those essentially tetrahedral Zn^2+^ ions (Bosch et al., 2014; Ravon et al., 2010).

It is possible to produce a wide variety of MOF structures to the extent that the geometry of each chemical constituent, size, and functionality can be modified (Furukawa et al., 2012) with unbeatable degrees of crystallinity and exceptional surface areas and pore volumes (Zhang et al., 2014; Liang et al., 2021). More tractable derivatives of the parent architecture of MOF are similar to autonomous motors due to their crystalline pore organization, which is structured by a combination of metal ions and ligands. In this setting, if a ligand has several possible conformations, the framework geometry will be difficult to predict, and several products can be formed. Of course, the formed complex structures are in close agreement with experimental values.

Using a condensation reaction between 4-(methoxycarbonyl) benzaldehyde and diphenylmethane, a Chicago team led by Lin synthesized the structure 5,15-di (p-benzoato) porphyrin (H_2_DBP). Functional alignment through dicarboxylate groups of the DBP ligand anchored the possibility of obtaining the framework DBP–UiO NMOF Hf_6_(*μ*_3_-O) 4 (*μ*_3_-OH)_4_ (DBP)_6_ by a solvothermal reaction between HfCl_4_ and H_2_DBP in DMF at 80°C (Lu et al., 2014).

Transition-metal coordination complexes are a broad class of supramolecular crosslinks used to engineer the mechanical properties of advanced structural materials. In this sense, the quality of this interplay can accentuate the instability of each metal-coordinate bond, which can be quantified in terms of a bond dissociation rate that is inversely related to bond dissociation time. These properties, combined, have suggested that metal-coordination bonds may be an emerging class of crosslink chemistries that could be used to tune static and dynamic mechanical properties in advanced materials (Khare et al., 2021). Grounded in the physical chemistry of metal-coordinate equilibrium dynamics, Cazzell and Holten-Andersen (2019) demonstrated that robust material crosslinking is thermodynamically favored if a minimum amount of metal is available during macromolecular self-assembly.

In 2012, Ikezoe et al. developed a hybrid biomimetic motor system consisting of an MOF and diphenylalanine (DPA) peptide. They used the MOF [Cu_2_L_2_ted]_n_ with a 0.75 nm sized pore, where L is 1,4-benzenedicarboxylate and ted is triethylenediamine. MOFs have the advantage of assembling small molecules into highly ordered pores and releasing them in a more isotropic direction by breaking the bonds of the framework. This is because of the behavior of the MOF in an energy-storing cell, which impacts peptide assembly inside the pores on the nanoscale order of the coordination framework. Consequently, due to the nature of the peptides, their assemblies can be reconfigured at a water/MOF interface and thus converted to fuel energy (Ikezoe et al., 2012).

Ferritin, an octahedral iron storage protein, was engineered in its C_3_ symmetric pores with tripodal Zn coordination sites and in accordance with an assembled framework structured by spherical protein nodes through metal–organic linker-directed interactions (Sontz et al., 2015). A detailed analysis of trends in the engineered metal-peptide strands shows that the entangled structures and the design of topological frameworks are inherent. In addition, we must not lose sight of the steric control of the peptide induced by ring opening and framework twisting (Inomata et al., 2021).

It should be mentioned that the biomimetic mineralization of MOFs impacts viruses. In this perspective, biotechnological encapsulation processes are used to protect healthy cells from the biological environment. The flexibility of the synergistic effects of MOFs on cells is noteworthy (Riccò et al., 2018). Because metal–ligand bonds dissociate following kinetics, the selection of the metal ion can even change the energetic effects of the material (Grindy et al., 2015). For example, conventional wound healing material loses its effectiveness because of this dissociation. The alternatives that provide greater security for more effective and faster healing, such as the use of MOFs, are very functional and have higher safety (Fu et al., 2020). An excellent study applied MXene (a family of 2D materials with the general formula of M_n+1_X_n_, M being an early transition metal and X being carbon or nitrogen) and a MOF-5 nanostructure to co-delivery of a drug and a gene to increase their bioavailability and interaction with pCRISPR. These nanomaterials were coated with alginate and chitosan to increase the surface potential. In this line of research, studies involving doxorubicin and its sustained release have made it possible to understand its cytotoxicity using HEK-293, PC12, HepG2, and HeLa cell lines, demonstrating acceptable cell viability at both low (0.1 μg mL^−1^) and high concentrations (10 μg mL^−1^) (Rabiee et al., 2021). Under these premises, a cooperative effect that optimized a nanocarrier agent consisting of a combination of MOF-5 and MXene, and chitosan and alginate, as exemplified in Figure 4, is explored in drug delivery and gene delivery applications (Rabiee et al., 2021).

Kashnik et al. proposed a reaction sequence generating a typical supramolecular ionic framework based on a tetracationic tetraphenylethene (TPE) and either a dianionic octahedral molybdenum cluster complex or a tetraanionic octahedral rhenium cluster complex. This reaction was carried out at 25°C by mixing the two precursor solutions. This approach focuses on octahedral metallic clusters of general formula A_n_ [M_6_L^i^_8_L^a^_6_] (A = alkali cation, M = transition metal, L^i^ = inner ligand, and L^a^ = apical ligand). The metallic scaffold M_6_ is covalently bonded to face-capping ligands (*μ*_3_‒L^i^) and is stabilized by six apical ligands La (Kashnik et al., 2024). In other studies in the biomedical field, we can find reviews about MOFs that detail specific structural requirements to have morphology to ensure biocompatibility and precise functionality in essentially all cases. For instance, 2D MOFs have been extensively studied as drug carriers for cancer applications as a consequence of the increased loading of chemotherapeutic drugs due to the MOF’s high specific surface area (Arun Kumar et al., 2021). Research has focused on the underlying antimicrobial mechanisms of MOFs and comprehensive evaluation methods of antimicrobial efficiency of MOF-based materials. Thus, a focused study on a specific subject where MOFs seem to be particularly effective encompasses next-generation MOF-based antimicrobial materials (Li et al., 2021).

The structuring of hybrid materials with unlimited functions is ensured by the provision of the new ideas linked above. The continuum of their properties can overcome the economic and technical hindrances arising in today’s technology (Chaouiki et al., 2023). In this circumstance, there is a great deal of space for customization on structuring MOF catalysts inherent to the tailoring ability of metallic building blocks and organic ligands. This can be exploited in the structuring of MOFs to be used as catalysts by the coordination unsaturation because the most reactive sites are often located in the edges and corners. In an excellent review, Gloag et al. (2024) laid the foundation to understand the co-catalytic metal–support interactions in single-atom catalysts (SACs) and the scaffolds and strategies for the development of improved catalytic materials. The article gives special attention to the different ways co-catalysis can influence the catalytic properties of an active site. One example is carbon-based composites (M–N–C) consisting of transition metals such as Fe, Co, and Ni and nitrogen, especially Fe–N–C, which can be effective for cathodic oxygen reduction reactions (Chen et al., 2019). These composites substitute for platinum-based catalysts (PGMs) by virtue of their low cost, high activity, and stability. The value of the MOFs widens toward the intrinsic catalytic activity that has been well outlined over the years in such a way that they have supported metallic nanoparticles and hosts for enzyme encapsulation (Bavykina et al., 2020).

In other studies, the encapsulation of the protein ubiquitin within self-assembled coordination cages has been realized. In this study, with the use of Pd^II^ ions (M) and bidentate ligands (L), M_12_L_24_ coordination nanocages are self-assembled around the protein (Fujita et al., 2012). In an unrestricted manner, comparing the conventional systemic treatment regimens and reducing systematic toxicity, the encapsulation of drugs within a single nanoscale drug delivery system can reduce systemic toxicity by suppressing the premature degradation and nonspecific interactions of drugs with normal tissues, ameliorating drug solubility, and prolonging circulation times in the blood (Demir Duman et al., 2022). By using this strategy, a team led by Forgan has engineered the synthesis of the Zr-MOF MOF-808, multi-functionalized through dual drug loading of floxuridine (FUDR) and carboplatin (CARB). Additionally, to enhance efficacy, a poly (acrylic acid mannose acrylamide) (PAAMAM) glycopolymer coating was utilized to reduce the side effects of chemotherapeutic treatment. Fine characterization showed that ∼10% (w/w) loading of CARB and ∼1% (w/w) loading of FUDR in MOF-808 nanoparticles was achievable (Demir Duman et al., 2022).

As anticipated, due to the directionality, MOF-based catalysts may be fabricated using functional groups containing organic ligands, including active heteroatoms such as carbon, oxygen, nitrogen, boron, and sulfur (Gopi et al., 2023). MOFs can be thought of as coordination compounds of amorphous crystalline constitution formed through the self-assembly of organic ligands with metal ions that exhibit a structured and punctual porosity and surface area (Chakraborty et al., 2021). The structure and topology can be designed using different chemical building units, as well as regulating synthetic parameters and post-synthetic treatments (Fonseca and Gong, 2022). From a general view, the topology of a molecule has a fundamental influence on its physical, chemical, and biological properties.

Until now, the metal–ligand coordination bonds have been used extensively in organizing molecular building blocks into diverse supramolecular architectures enhancing 1D, 2D, and 3D networks, usually known as coordination polymer (CordPs) or MOFs. In coordination polymers, metal ions enhance the design of desired topological structures and are attractive to researchers. For instance, the strong metal‒ligand (M‒L) bonds can be expanded to the area of crystal engineering (Batten et al., 2016) in the context of the liquid and solid aspects present in CordPs and MOFs. Coordination chemistry and reticular design principles offer unlimited possibilities for extending the structural diversification and properties of CordPs and MOFs (Ma and Horike, 2022). Furthermore, the structural role can be further complemented by added functionality to produce a new electronic control through non-bonding interaction. The chemistry of CordPs and MOFs is intimately linked with the inorganic nodes with metal ions or inorganic polynuclear clusters known as SBU, which are linked with an organic linker through coordination bonds.

The consistent physicochemical properties of MOFs make them particularly interesting for a wide radius of applications such as adsorption (Jamshidifard et al., 2019), nonlinear optics (Ma et al., 2023), catalysis, gas storage and separation, air filtration, sewage treatment, sensing, and energy storage (Cai et al., 2021). MOFs with such special characteristics are also known as porous coordination polymers (PCPs). They can create stabilizing microenvironments for some biomolecules to improve their performance against perturbation conditions but also promote the separation and recovery of biomolecules from some industrial products (An et al., 2019; Doonan et al., 2017). The structural richness based on metallic nodes and organic functional linkers gives them varied structural richness with multiple and selective interactions. Several studies point toward strategies for improving the processability of MOFs, especially through the combination of MOFs with other materials to form macro-MOFs with enhanced processability, high flexibility, and excellent mechanical properties (Cheng et al., 2020). The improved mass diffusion properties of MOFs, together with their robust single-crystalline nature, endow them with superior catalytic activity and recyclability for bulky-molecule reactions (Shen et al., 2018). In this environment, Rosi et al. identified the principal topological possibilities of a variety of MOFs, showing that MOF-74, also called CPO-27 (the coordination polymer of Oslo), is one of the most promising MOFs with stable architectures and high porosity. The authors reported on a set of MOFs with polytopic carboxylates containing Zn, Pb, Co, Cd, Mn, or Tb in their structure (Rosi et al., 2005). In another instance, they discuss the synthetic approaches and their respective advantages for mixed metals in MOF-74 frameworks through diverse paths of ligand extensions to configure the pore size, surface area, and functionality. A convenient method for preparing mono- and bisferrocenylphosphinic acids has been developed by the group of Khrizanforov, establishing a structural reference on the coordination of phosphorus atoms and reaction conditions of bisferrocenylphosphinates. These structural ligands have an impact on the formation of coordination polymers and their inherent properties (Shekurov et al., 2023).

To conclude this section, some studies have addressed the structural complexes of carbohydrates that provide well-defined data about the secondary structures, separated by energy barriers, and strongly suggest chemical strategies to stabilize a particular conformer. Consequently, the directed design of oligosaccharide chains is feasible to tune glycan aggregation and program supramolecular assembly (Djalali et al., 2024). In this regard, a detailed understanding of the κ-carrageenan is reported by Mezzenga and collaborators, providing important information about the modulation of the forms of single helices when κ-carrageenan is subjected to potassium ions to build a secondary structure, further folding into single-chain supercoiled strands to form a tertiary structure, and then forming side-by-side rigid superstrands with other chains to set up a quaternary structure. This is a fundamental tool for enhancing a branched supramolecular physical network with local anisotropic orientation (Diener et al., 2019).

## 5 Electrochemical synthesis of MOFs and their related specific architecture

In the 18 years since the introduction of anodic dissolution by BASF, electrosynthesis has matured. Electrosynthesis is related to anodic dissolution, where the oxidation of an electrode functions as a source of metal ions essential for a specific MOF formation (Al-Kutubi et al., 2015). Anodic dissolution, supported by gentle reaction conditions, allowed the continuous synthesis of compact homogeneous MOF thin films within a short period of time. Additionally, an attempt has been made to combine approaches to the real-time monitoring of electrodeposition, allowing continuous control over the synthesis and subsequent scalability (Varsha and Nageswaran, 2020). Certainly, in direct electrosynthesis, such as anodic dissolution and reductive electrosynthesis, the desired MOFs are formed directly on the electrode surface by an electrochemical reaction. By using indirect electrosynthesis, MOFs are generated via electrophoretic deposition, galvanic displacement, or self-templated synthesis (Udourioh et al., 2022). MOF-based photoelectrochemical (PEC) applications can be applied to alcohol oxidation, water oxidation, water splitting, CO_2_ reduction, solar cells, water remediation, and sensors and biosensors (Dashtian et al., 2021).

It is noteworthy that the MOF thin films (MOFTFs) have been developed using spin-coating, seeded growth or secondary growth, electrophoretic deposition, Langmuir Blodgett, layer-by-layer (LBL) deposition, evaporation induced crystallization (EIC), gel-layer synthesis, chemical vapor deposition (CVD), dipping layer by layer (DLBL), pump layer by layer (PLBL), hydro (solvo) thermal, spray layer by layer (SLBL), flowing layer by layer (FLBL), and anodic and cathodic electrochemical deposition (ECD) (Dashtian et al., 2021). It is worth mentioning that the host–guest interactions at the supermolecule level have an influence on charge transport, achieving precise control over the conductance. This influence, and taking advantage of supramolecular chemistry, suggests the possibility of creating novel techniques of supramolecular polymerization to generate a range of catalytic systems (Chen and Fraser Stoddart, 2021).

Exploring synthetic methods to produce MOFs is increasingly tinged with innovation; they can be produced using hydro (solvo) thermal, microwave synthesis, electrochemical synthesis, and direct precipitation. It goes without saying that the metallic component is of utmost importance in providing specific active sites for catalysis and adsorption (Bai et al., 2024). Ni-based MOFs can form a film on nickel foam; that is, a layer of Ni_3_(BTC)_2_ can be formed by an electrochemical process at a constant voltage. Likewise, the effect of solvent, voltage, and reaction time, as well as the electrocatalytic efficiency of the NiBTC/Ni system, on the hydrogen evolution reaction (HER) has been fully studied (Jabarian and Ghaffarinejad, 2019). Additionally, it is reported that the formation of Ni_3_(BTC)_2_ from NiBTC was obtained by dissolving 15 mmol of BTC and 33 mmol of MTBS in 100 mL of different ratios of EtOH/H_2_O (Jabarian and Ghaffarinejad, 2019).

The patterned Ni organic framework was developed by Vinesh et al., who grafted TMA with Ni^II^ ions and the fractional cation replacement of Ni^II^ with Co^II^ in a paddlewheel organization of a Ni MOF (Vignesh et al., 2022). The bimetallic MOF carbonized at 500°C generates the homogeneously spread NiO/Co_3_O_4_ hollow structures with hierarchical carbon architectures, such as NiO/Co_3_O_4_/C. Betal oxide/carbon nanocomposite-loaded biodegradable corn starch bags (BCSBs) are employed as electrochemical probes for enzyme-free glucose detection. The major impetus for using NiO/Co_3_O_4_/C as a means of diagnosing glucose came from the group of Kumar (Vignesh et al., 2022) in a substantial article. In addition to using density functional theory (DFT) studies and the impact on glucose electrooxidation reaction (GEOR) kinetics, the article comments on diversified electrochemical characterizations.

There are continuing efforts to correlate, predict, and understand the feasibility of replacing the traditional, toxic, fossil fuel-derived solvents in electrochemical MOF synthesis. If this is feasible, it would be a real advance in the use of cleaner alternatives. Bhindi et al. showed the potential benefits by making use of γ-valerolactone (GVL) and dihydrolevoglucosenone (Cyrene^TM^) to electrochemically synthesize a wide range of MOFs with either Cu, Zn, or Co as the metal component and either 1,3,5-benzenetricarboxylic acid (BTC), imidazole (IM), benzimidazole (bIM), 2-methylimidazole (mIM), or 2-ethylimidazole (eIM) as the organic linker component. However, structures become less stable with higher porosity, indicating that the larger biosolvent molecules may also affect the synthesis of MOFs (Bhindi et al., 2023). In their 2023 article, Zhang, Fransaer, and coworkers made interesting suppositions about MOF films by controlling the supersaturation of molecular building units of MOFs during anodic electrodeposition. The characteristic patterns are discussed by increasing the pH of the electrolyte to facilitate the deprotonation of linkers and thus to design a film of HKUST-1 with morphologies as varied as octahedral, cuboctahedral, and cubic. Considering the electrochemical quartz crystal microbalance (EQCM) technique combined with *ex situ* SEM measurements, the authors suggest that the supersaturation during crystal growth is bound to affect the crystalline morphology (Guo W. et al., 2023). Another approach to the use of electrochemistry is the synthesis of the complex CoNi-ZIFs@Ag@NF based on CoNi-ZIFs and Ag NPs. This complex is a substrate potential for SERS monitoring of tetracycline in food and the environment (Zhao et al., 2023).

Hiroata et al. have shown that control of crystal morphology can result in pillararene molecule stacking with the cationic species of the electrolyte. It is possible that the host–guest complex precursor releases the guest molecules during the oxidative transformation because the obtained pillarquinone is an electron-deficient macrocycle. Moreover, manipulating the morphology by varying the solvent composition is a key factor (Hirohata et al., 2024).

There are clear advantages to producing MOF materials in the form of thin films with perfectly defined geometries (Araújo-Cordero et al., 2024). These geometries have been finely developed over time and comprise solid and hollow spheres, prisms, rods, wires, and dendrites. There have also been great advances to increase the active sites in the catalyst for the electrochemical synthesis of MOFs.

## 6 Switchable MOFs

A MOF perfectly designed to present adaptability in its conformation and, therefore, modify its pore size must be constituted of chemical entities that have the property of switchability (sometimes called flexibility phenomena). Switchability implies a stepwise specific response (solid-state phase transition) at a well-defined chemical potential (Miura et al., 2023). Engineering 4D materials is equivalent to controlling activation barrier materials, a scientific field that has been moderately explored. The ON–OFF switchable function is realized by applying electrical signals (Sterin et al., 2024).

Note the existence of a response spatiotemporal response governed by an activation barrier, reflected in the gate opening pressure (P_APHM_) and one activity of a pore opening stimulating guest molecule (P_stim_). In truth, the ensemble-based switching can be represented by the rate constant k with Δp = P_stim_ – P_APHM_ (Miura et al., 2023).

Switching process criteria lead to key strategies in the adsorptive and desorptive control of materials. Compounds that perform well as switches in solution may encounter difficulties in the solid state owing to a greater restriction of movement in the solid state. An additional reason for using switching processes is present in photoresponsive molecules that exhibit potential use as sensors, switches, and memory and optical data storage media. Clear examples are the organometallic molecules widely investigated and used in technologies such as organic light-emitting diodes (OLEDs), fluorescent probes, and laser dyes (Bigdeli et al., 2020).

To talk about multi-photon absorption (MPA) is to detail the nonlinear optical (NLO) properties for applications as varied as telecommunications, photonics, and biomedicines. It is not surprising that MOFs with benefits such as MPA have been designed consisting of zirconium- and hafnium-oxoclusters and featuring a chromophore linker based on the tetraphenylethene (TPE) molecule showing a considerable two-photon absorption with cross-section values up to 3600 units Göppert–Mayer (G-M), (1 G-M = 10–50 cm^4^sPhoton^−1^molecule^−1^) (Medishetty et al., 2017). With this notion, the design of MOFs for MPA applications follows several criteria for organic or organometallic NLO chromophore molecules. One criterion is the focus of molecular dipole or multipolar structural units for an enhanced polarization of the charge distribution and other factors. Equally relevant are the presence of a planar structural pattern with long pi symbol-conjugation, the increase in fluorescence quantum yield, the narrow one-photon and two-photon absorption bands, and the alignment and high packing density associated with the chromophore with NLO properties. The electrophilic charge polarization criterion has been generally appropriate in two series of MOF Zr-based nodes. There are two basic approaches. In the first, an isostructural Hf-analog is established; subsequently, electron-withdrawing TFA-bonded structures are modulated in order to stabilize a charge transfer (Medishetty et al., 2017).

An interesting idea about switchable MOFs is proposed by Aprahamian, who explains that an existing switch in the MOFs is understandable by their free volume, especially if they are used as side groups, but less so if they are used as part of the framework. To apply this, it is necessary to know the feasibility corresponding to careful crystal engineering, which is required to allow the framework to freely switch its geometrical shape. This is related, in turn, to the architecture of the types of molecules that show that the function and dynamic motion are attainable as a diversification of systems that can be used as switchable crystals (Aprahamian, 2020). It can thus be seen that Cu-MOF-SCH_3_ displays an especially switchable structure for the separation of a CH_4_/N_2_ mixture, which is assumed to be due to the structural change through equilibrium adsorption. This allows considerable separation of CH_4_/N_2_ at 298 K and 1.0 bar (Chang et al., 2023). Some excellent experiments have clarified and confirmed a hypothesis about the metastable states upon sorption in mesoporous MOFs and the appearance of a liquid-like condensed phase within mesopores triggering a structural transition in the system DUT-49(Cu) accompanied by the negative gas adsorption (NGA) phenomenon (Walenszus et al., 2023).

Recent leading studies on the energy barriers that are the criterion that reveals kinetic control emphasize that it can arise from a stability framework coupled to the physics of the guest molecules with characteristic barriers of fluid nucleation, diffusion, and adsorption processes (Miura et al., 2023). Progress in measuring the transformation time of a switchable MOF has consequently been a tedious task. This weight of evidence supports the cyclic variation of the applied electric potential on an electrode modified with the ZIF-8 MOF as a prototypical model that is labile under acidic conditions due to protonation of the Zn-N ligand bond and can reversibly activate and inhibit the payload release. In general, this is the case because the structure of ZIF-8 has a three-dimensional pore network composed of sodalite cages as large as 13.4 Å, interconnected by smaller 3.4 Å apertures (Sterin et al., 2024).

Negatively charged nitrogen-containing chemical entities have been exploited for the magnetic switching of a MOF, which is composed of cyanoheterometallic units of Fe and Ag, that is, [Fe (1,6-naphthyridine)_2_(Ag(CN)_2_)_2_]. The presence of paramagnetic Fe^III^, either in low-spin (LS, S = 0, no unpaired d-orbital electrons) or high-spin (HS, S = 2, four unpaired d-orbital electrons) states, impacts the detectability of nitrogen vacancy (NV‒) in the form of optically detected magnetic resonance (ODMR) and magnetic modulation (MM) (Flinn et al., 2024).

On the other hand, the replacement of silica gel and zeolite with MOFs has been carried out by Askalany and collaborators for desalination. CPO-27(Ni) MOF was used in a series of cycles of desalination, switching times, and different cooling water temperatures to identify the optimal conditions and achieve the maximum value of specific daily water production (SDWP) and gained output ratio (GOR) of the cycle (Askalany et al., 2021). In another case, the coordination between Cu^+2^ ions with the oxygen of the sulfonic group and the amino group of taurine has been studied. Under this premise, the PLL-GQDs@UiO-66@NH_2_ MOF fluorescent nanoprobe may be used for taurine biosensing in biological fluids with high specificity (Nangare et al., 2024).

Another model established by the group of Youssef is linked to the volumetric magnetization of the crystalline mixture composed of poly 3-hexylthiophene-2,5-diyl (P3HT) and the fullerene compound phenyl-C_61_-butyric acid methyl ester (PCBM). The P3HT:PCBM crystal blend obtained shows soft magnetic behavior with a remanent magnetization of 6.4 memu/cm^3^ and a magnetic field of 9.4 Oe. It is inherent that the crystalline P3HT:PCBM complex presents a trace quantity of pinning sites that stimulate the soft magnetic response, improving switchable magnetics (Newacheck et al., 2024).

Dynamic machinery of the MOF FTR-P2 (belonging to the family of frustrated trigonal rotors) has been assessed by Perego et al. These molecular dynamics may be due to a guest-stimulated flexibility, and it may be indicated that FTR-P2 comprises a molecular dynamic of the set of bCP (synthesized by assembly bicyclo [1.1.1]pentane-1,3-dicarboxylate and azpy = 4,4′-1,2-diazenediylbispyridine), metal nodes, and azo pyridyl pillars (Perego et al., 2024).

**FIGURE 5:**
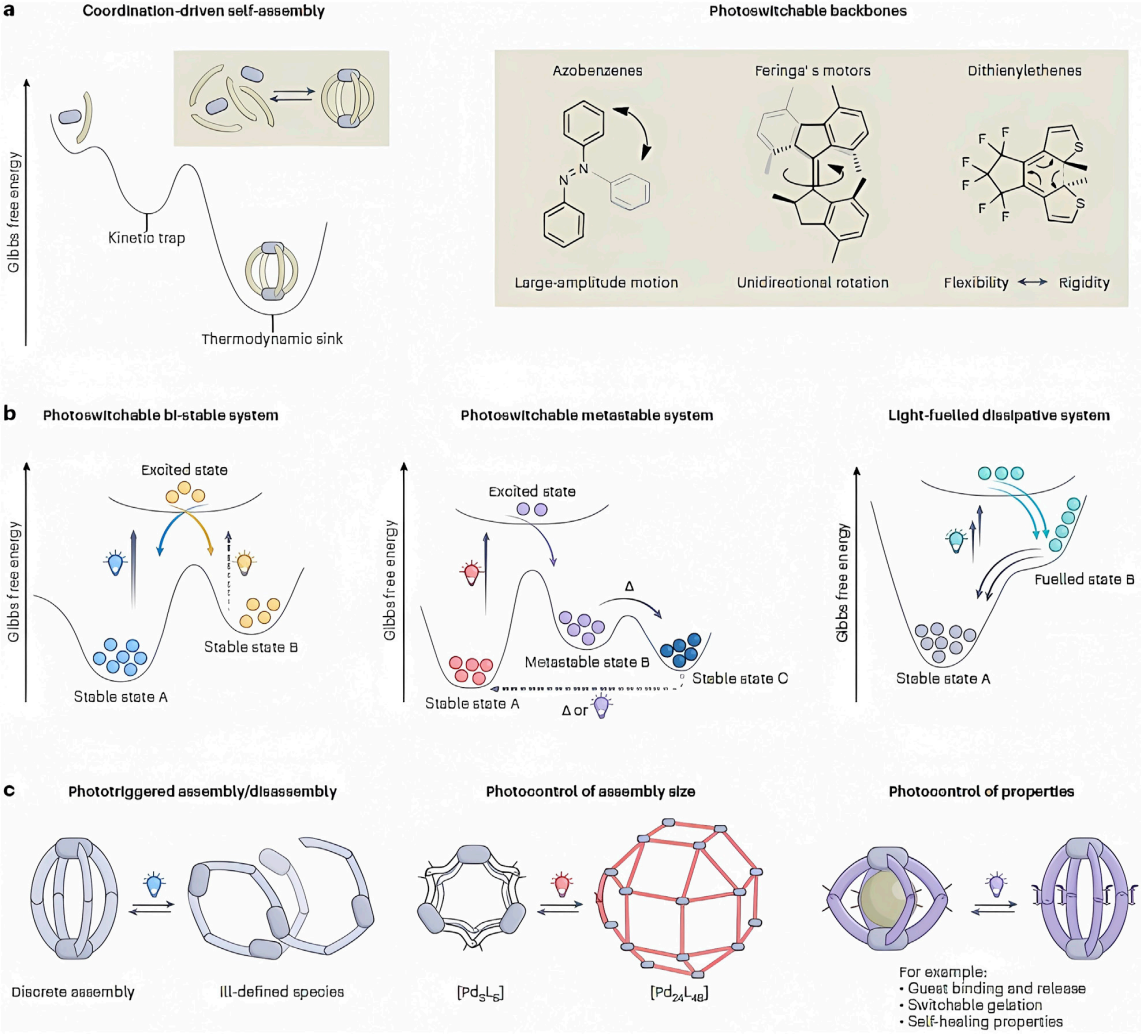
Photoswitchable performance into metal-mediated assemblies. **(A)** On the right side, we can see that metal-mediated self-assembly of bridging ligands with photoswitchable backbone leads to stimuli-responsive coordination cages. **(B)** Three scenarios as a function of back-switching kinetics. On the left: switching between two stable states. In the middle part: light-triggered preparation of a metastable state, followed by thermal relaxation into another stable state. On the right: continuous irradiation is required to maintain the population of the high-energy isomer in an out-of-equilibrium system. **(C)** Examples of the effects of light-responsive backbones include the integrity, structure, and properties of metal-mediated assemblies. Adapted from Benchimol et al. (2024).

Through electronic excitation, it is feasible for a system to tame the energetic barrier and an intramolecular reaction that can be a double-bond isomerization or controlled electrocyclic bond shifts. Configurational molecular reestablishment can be obtained either by the application of a different wavelength or by thermal relaxation. However, when a metal atom is involved, as indicated in Figure 5A, everything depends on the coordination dynamics and the kinetic stability with respect to thermal switching, as illustrated in Figure 5B. Of course, the energy barrier increases as the molecular complexity increases, as it does in stable geometric systems. All of this can be observed in Figure 5C (Benchimol et al., 2024). In this regard, a detailed analysis of trends in the thermodynamics of a system that converts light into chemical energy photostationarily out of equilibrium has been provided by Gemen et al. (2023).

The comparable trend between the double dipolar chromophores can be overviewed by photogenerated radicals, which permit the *in situ* switchable output on both the isotropic and anisotropic states, and by fluorescence emission (Xiong et al., 2024).

**FIGURE 6:**
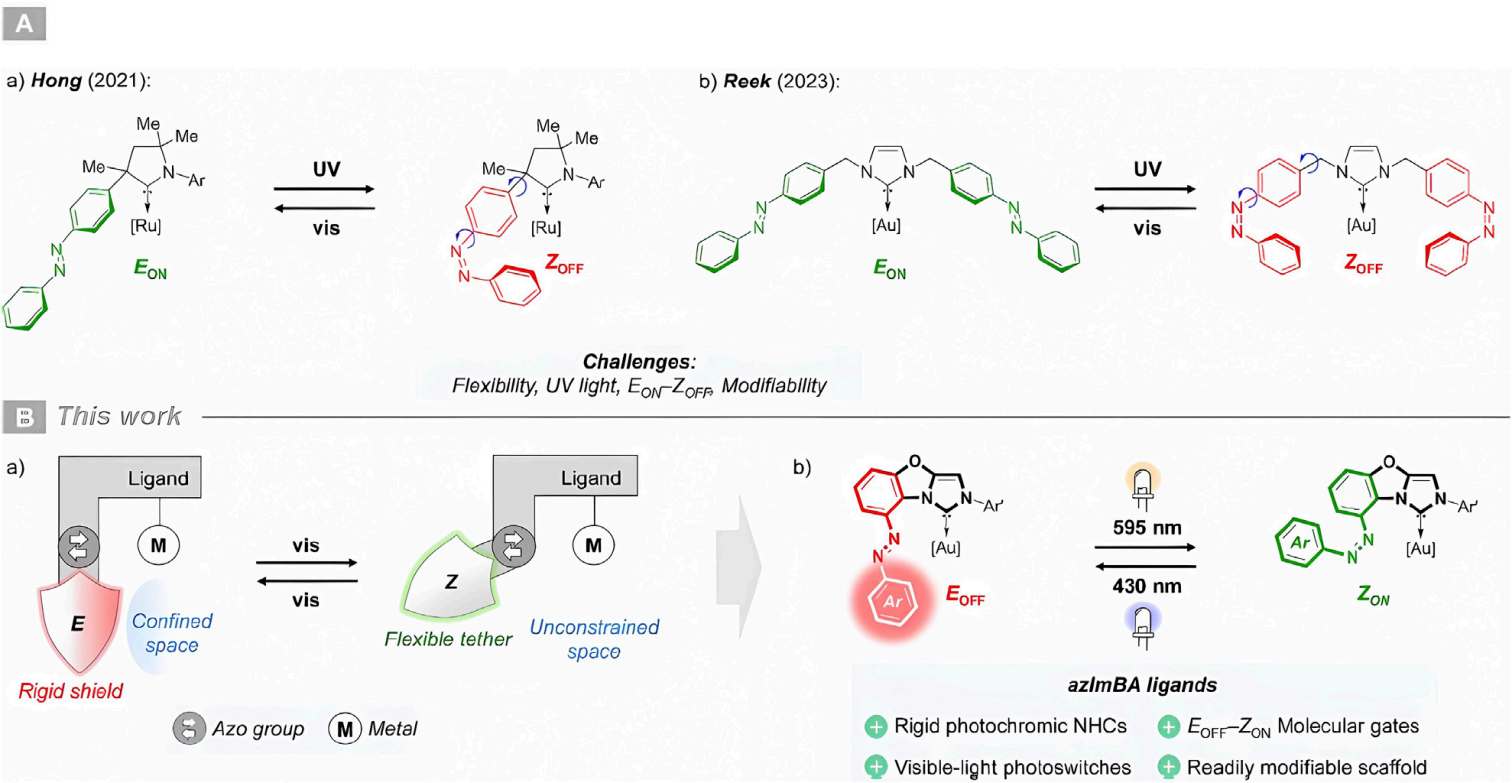
**(A)** In previous studies: (a) Azobenzene-based CAAC and (b) NHC for steric photomodulation of activity. **(B)** In Rölz, Butschke, and Breit’s work: (a) Photoswitchable confinement in a metal complex, and (b) development of azImBA ligands applied to Au^I^ chemistry. Photoswitch units are highlighted in green for the high activity/ON state and in red for the low activity/OFF state. Adapted from Rölz et al. (2024).

On the other hand, the electronic properties of spiropyran-based MOFs with Cu nodes have been predicted using molecular dynamics simulations achieving on–off switching. The existence of electron-donating functional groups alters the energy, and the occupation of the frontier orbitals influences the electronic coupling between the linkers in the MOF (Mostaghimi et al., 2023). Other related examples include the photochromic properties of lanthanide-structured MOFs and the photoresponsive moiety 4,4′-azopyridine that seek to establish a photophysical material (Martin et al., 2024). The same can be indicated from the photochromic studies that have recently been addressed by Rödl et al. (2024) involving the fluorinated azobenzene (Fx-AZB) compound as a non-covalently attached guest molecule within UiO-66. Several studies also detail a photoswitchable *N*-heterocyclic carbene (NHC) derived from tricyclic imidazo [5,1-b]benzoxazol-1-ylidene designed to enable visible-light-switchable reactivity of metal complexes as a promising platform for the progress of artificial catalytic systems (Rölz et al., 2024). The photoswitching of catalytic activity in ring-closing metathesis can be visualized in Figure 6A. Likewise, this photoswitching is due to the effect of light allowing the alternation between strongly confined L-shaped *E*-rigid states and an open and flexible *Z*-state, as indicated in Figure 6B.

### 6.1 Metalloporphyrinic structures

**FIGURE 7:**
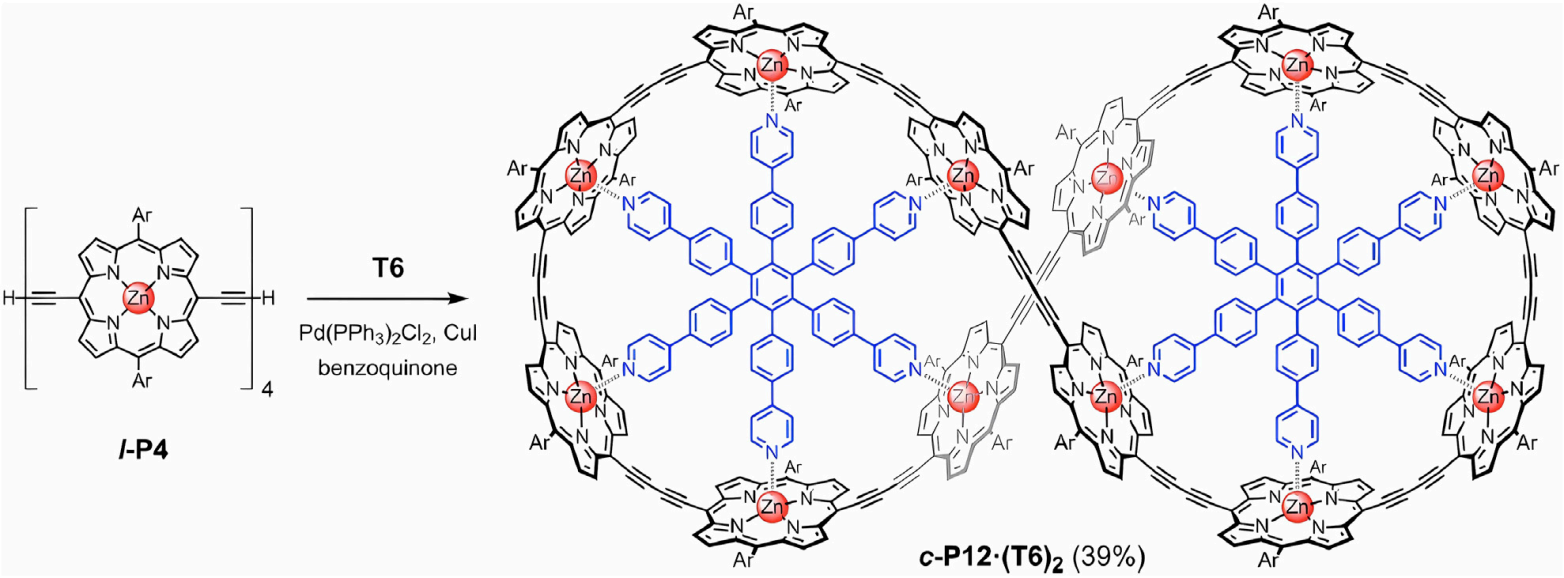
Vernier templated synthesis of a 12-site macrocycle (*c*-P12). The yield of a figure-of-eight complex (*c*-P12·(T6)_2_) is 39% for the case with 3,5-di-tert-butylphenyl. Adapted from Bols and Anderson (2018).

Other widely used supramolecular systems based on imine groups are the supramolecular porphyrinic cages under schemes immersed in MOFs that form dynamic complexes in the domain of reversibility. Some observations have reported yields and clear evidence of secondary products along with the target primary products (Nisa et al., 2023). At this point, Cook et al. (2017) gave an excellent survey of the porphyrins and multiporphyrins. They also focused on its triclinic or monoclinic symmetry. Thus, although this article is extremely significant, it is not a trivial exercise to compare the construction of metalloporphyrins; contextualizing, one pyridine binding site is able to successfully coordinate with one metalloporphyrin center (Bols and Anderson, 2018). This is the case of, as indicated in Figure 7, the linear *l*-P4 units that can be coupled together covalently to allow an 8-shaped complex, in particular, *c*-P12·(T6)_2_. Alternatively, a template with pyridine produces a free macrocycle *c*-P12 (Bols and Anderson, 2018).

Porphyrins are an example in which the aromatic properties in pyrrole moieties are associated with a joint molecular property in the center of the macrocycle. In the metal complexes, all four pyrrole rings are incorporated into the aromatic system. Meanwhile, in the case of dianions, the pi-electronic structure is more strongly localized on the central part, which can be considered as an 18 π-electronic species plus four C_2_H_2_ bridging groups (Cyrañski et al., 1998). Specifically, the impact on the CC and CN bond lengths in porphyrins is perturbed by exocyclic substitution and coordination with a metal cation.

Because of the relative aromaticity of porphyrin, it can support a range of oxidation states, including the interactions where the redox process occurs in the metal ligand. Although it has been shown that the strength of the porphyrinic ring current can be measured by the effect of its chemical modification in applications such as molecular electronics, molecular machines, catalysis, therapy, and surface engineering, free-base porphyrins in contrast to metalloporphyrins can provide an added synthetic advantage as long as the central metal atom can act as an acceptor site and enable a higher level of complexity by axial coordination. Peculiarly, Zn^II^-metalated porphyrins acquire the square pyramidal geometric configuration by the formation of the axial bonds; that is, the directionality of the Zn-N bond may well be a great advantage (Percástegui and Jancik, 2020).

A variety of methods have been developed for the synthesis of metalloporphyrins. They have been evaluated using proton conduction by complex electrochemical impedance spectroscopy (EIS). Significantly, different types of structural units can be introduced systematically with impact on the final properties; for example, [H (bipy)]_2_ [(MnTPPS) (H_2_O)_2_]·2bipy·14H_2_O, where bipy is 4,4′-bipyidine and TPPS^4−^ is the meso-tetra(4-sulfonatephenyl) porphyrin, shows a zig-zagging water chain between the sulfonate groups of the porphyrin and presents a conductivity of 1 × 10^−2^ S·cm^−1^ at 40°C and 98% relative humidity, which is an impressive value (Fidalgo-Marijuan, Ruiz de Larramendi and Barandika, 2024).

A conjugated imine-linked porphyrin–homopolymeric COF prepared by a Schiff base condensation of 5,10,15,20-tetrakis (4-formylphenyl)-21*H*,23*H*-porphyrin, namely TFPP, and 5,10,15,20-tetrakis (4-aminophenyl)-21*H*,23*H*-porphyrin, namely TAPP, has been successfully obtained. Consenting to the same pathway, regioregular multimetalloporphyrin COFs with two different metal-center combinations, including Zn and Cu typed as *por*-COF-ZnCu, and Zn and Ni typed as *por*-COF-ZnNi, have been synthesized for NLO applications. Intensity-dependent NLO switching behavior was observed, which is a desirable factor for optical switching and optical limiting devices (Biswal et al., 2019). Even self-assembly properties of amide-based porphyrins, with chemical functionalities C=O or centered N‒H, have been synthesized by Weyandt et al. (2021), revealing that these molecular designs support photo-optical attributes.

The characteristic patterns of the design of photocatalyst molecular schemes for photodegradation techniques are discussed by Shee and Kim (2024). In this context, the geometries, hyperfine structures, and relative stabilities of MOFs are advantages due to their coordination architectures, functional active sites, and permanent void spaces inside the pores (Shee and Kim, 2024).

Upon formation of tetrameric supramolecular architectures prepared through the reaction of trans-dihydroxo-[5,15-bis(4-pyridyl)-10,20-bis(phenyl) porphyrinato]tin^IV^ and trans-dihydroxo-[5,15-bis(4-pyridyl)-10,20-bis(4-tert-butylphenyl) porphyrinato]tin^IV^ with Re(CO)_5_Cl (pentacarbonylchlororhenium(I)), thermodynamic stability studies have been promoted in the degradation of Eriochrome Black T dye (Shee and Kim, 2024).

We have brought to light the potential of porphyrin as a key to new technologies. Clearly, there is much to be said in this regard, which is amplified when a chemical modification of the porphyrin and diverse coordination with metallic ions exist.

## 7 Summary, conclusion, and outlook

Through this review, an attempt has been made to bring together old and new ideas regarding the association of supramolecular chemistry with MOF structures covering an entire molecular spectrum adapted to circumstances and trends. Classical MOF discussions have brought together theoreticians and synthetic chemists. In addition, any attempts to understand supramolecular structures promote further interdisciplinary interaction by covering larger and larger self-assembly systems and, eventually, structures under strictly planned molecular design. The chemistry and physics of supramolecular structures have developed into the science and technology of electronic materials. However, even if the reader tends to confine their view to the fundamental aspects of supramolecular structures, extended self-assembly systems create challenging problems for discussing MOFs and supramolecular structures and their practical and specific advances. The other important aspect is that while being concerned with the conjugation of MOFs and supramolecularity, one should not restrict attention to intramolecular aspects. Properties such as supramolecular chemistry in a confined space, relationships of MOFs with supramolecular chemistry, electrochemical synthesis of MOFs, and switchable MOFs, which are regarded as being characteristic of molecules whose origin is the formation of assembly systems to obtain supramolecular MOFs, cannot be understood when attention is focused upon the behavior of a single molecule or small isolated molecules in dilute solution. This is the reason that classic discussions regarding MOFs have brought together theoreticians and synthetic chemists.

Quantitative prognostics emerging from very particular lines of research have also provided a strong challenge both to theory and experimentation. Hence, many examples still await both vigorous theoretical examination and experimental verification, and the stimulus to achieve these goals promises to continue to advance chemical knowledge. We believe that great progress is still to be expected in the coming few years, taking advantage of the significant influence of the chemistry of the MOFs and supramolecularity on porphyrin and its structural variants.

## Glossary

APHM: Adsorption pressure at half maximum uptake
APT: Atom probe tomography
AZPY: 4,4′-1,2-diazenediylbispyridine
bCP: Bicyclopentane
BCPs: Block copolymers
BCSBs: Biodegradable corn starch bags
bIM: Benzimidazole
bipy: 4,4′-bipyidine
BTC: 1,3,5-benzenetricarboxylic acid
CARB: Carboplatin
CDC: Constitutional dynamic chemistry
CDSA: Crystallization-driven self-assembly
CPs: Conducting polymers
COFs: Covalent organic frameworks
CordP: Coordination polymer
CPO: Coordination polymer of Oslo
CVD: Chemical vapor deposition
DFT: Density functional theory
DLBL: Dipping layer by layer
DMF: *N*,*N*-dimethylformamide
DPA: Diphenylalanine
DXTA: 2,5-dihaloterephthalate
ECD: Electrochemical deposition
EIC: Evaporation-induced crystallization
eIM: 2-ethylimidazole
EO: Ethylene oxide
EQCM: Electrochemical quartz crystal microbalance
E-SPN: Electrically conducting supramolecular polymer network
FLBL: Flowing layer by layer
FTR: Frustrated trigonal rotors
FUDR: Floxuridine
GEOR: Glucose electrooxidation reaction
G-M: Göppert–Mayer
GOR: Gained output ratio
GVL: γ-valerolactone
HER: Hydrogen evolution reaction
**H**_**2**_ **DBP**: 5,15-di(p-benzoato)porphyrin
IM: Imidazole
LBL: Layer by layer
**Me**_**3**_ **mpba**: *N*,*N*′-2,4,6-trimethyl-1,3-phenylenebis(oxamate)
MIL-140A: Framework isomer of UiO-66
mIM: 2-methylimidazole
MM: Magnetic modulation
MOFs: Metal–organic frameworks
MOFTFs: MOF thin films
MOGs: Metal–organic gels
MPA: Multi-photon absorption
MTBS: Tributyl methylammonium methyl sulfate
NGA: Negative gas adsorption
NHCs: N-heterocyclic carbenes
NLO: Nonlinear optical
NMOF: Nanoscale metal–organic frameworks
PAAMAM: Poly(acrylic acid mannose acrylamide)
PAG: Polyalkylene glycol
PCBM: Phenyl-C_61_-butyric acid methyl ester
PCPs: Porous coordination polymers
pCRISPR: Plasmid clustered regularly interspaced short palindromic repeats
PEC: Photoelectrochemical
PEDOT: PSSPoly(3,4-ethylenedioxythiophene):poly(styrene sulfonate)
PLBL: Pump layer by layer
PLL: Poly-L-lysine
PO: Propylene oxide
**Ru(bpy)**_**3**_: Tris(bipyridine)ruthenium(II)
SBUs: Secondary building units
SCCs: Supramolecular coordination compounds
SDWP: Specific daily water production
SERS: Surface-enhanced Raman scattering
SLBL: Spray layer by layer
TAPP: 5,10,15,20-tetrakis(4-aminophenyl)-21H,23H-porphyrin
Ted: Triethylenediamine
tetraPEG: Tetra-arm poly(ethylene glycol)
TFA: Trifluoroacetate
TFPP: 5,10,15,20-tetrakis(4-formylphenyl)-21*H*,23*H*-porphyrin
TMA: Trimesic acid
TPE: Tetraphenylethene
**TPPS** ^**4−**^: Meso-tetra(4-sulfonatephenyl) porphyrin
TTF: Tetrathiafulvalene
UiO‒MOF: Featuring UiO-66, UiO-67, UiO-68, and UiO-69
UiO-66: Zirconium-based MOF (stands for University of Oslo)
ZIF: Zeolitic imidazolate framework.
